# Clinical characteristics and cancer-specific survival analysis of double primary cancer patients with lung cancer as the first primary cancer

**DOI:** 10.1097/MD.0000000000030173

**Published:** 2022-08-26

**Authors:** Bin Hu, Wanjiao Chen, Ningjie Xu, Jiarong Lv, Shifang Sun, Yifeng Mai

**Affiliations:** a School of Medicine, Ningbo University, Ningbo, China; b Department of Geriatrics Medicine, the Affiliated Hospital of Medical School, Ningbo University, Ningbo, China.

**Keywords:** double primary cancer, lung cancer, prediction model, prognosis

## Abstract

The objective of this study is to explore the prognostic factors of double primary cancer patients with lung cancer as the first primary cancer (FPC). The Surveillance, Epidemiology, and End Results (SEER) database is a database established by the National Institutes of Research for cancer registration purposes, which collects relatively complete demographic characteristics and clinical data for assessing the epidemiological characteristics of cancer worldwide. Clinical data on patients with a clear histopathological diagnosis of double primary with lung cancer as the FPC were identified and collected from the SEER database from 2010 to 2015. Survival curves were plotted by Kaplan–Meier survival analysis. Independent prognostic factors of patients were analyzed by COX proportional risk model. Clinical data were collected from a total of 9306 patients, including 6516 patients in the modeling group and 2790 patients in the validation group. When we retrieved that the FPC was lung cancer, we found that the most common site of the second primary cancer was located in the respiratory system (54.0%). In addition, the most common site of first primary lung cancer in patients with double primary cancer was the right upper lobe (33.3%). A total of 14 independent prognostic factors were included, and the constructed survival nomogram had high accuracy and clinical applicability. The nomogram established in this study can help to raise awareness of clinical workers and the importance of such diseases, and guide the treatment and follow-up strategies.

## 1. Introduction

Lung cancer is one of the most common malignant tumors in the world, with the highest incidence and mortality rates.^[1]^ With the decline in the number of people who smoke, the development and application of early lung cancer screening programs and the development of new targeted drugs, the 5-year survival rate of lung cancer is now higher than before, nonetheless the prognosis remains incredibly poor.^[1,2]^ One of the major reasons for the poor prognosis of lung cancer patients is the existence of posttreatment long-term complications.^[3]^ Currently, there is a relative lack of information related to long-term survival complications and survival prognosis of lung cancer patients in China.

Multiple primary cancers, as a category of long-term survival complications of cancer, are mainly manifested by the simultaneous or sequential appearance of 2 or more unrelated primary malignancies in the same patient.^[3]^ With the innovation of cancer-related detection technology, the incidence of multiple primary cancers is increasing. However, when encountering such patients, clinical workers often misdirect them as metastatic lung cancer and lose confidence in their prognosis, and mostly focus on palliative and supportive treatment. Currently, the diagnosis and treatment of multicellular cancers remain a major challenge.

The Surveillance, Epidemiology, and End Result (SEER) database collects relatively complete demographic characteristics and clinical data for assessing the epidemiological characteristics of cancer worldwide.^[4]^ The American Joint Committee on Cancer tumor node metastasis (TNM) staging currently used has limitations in predicting the prognosis of multiple primary cancers, and the nomogram is being widely used as a tool for individualized cancer prognosis prediction. The use of nomogram to quantify and predict relevant factors can better reflect the impact of cancer and treatment-related information on the survival of patient organisms.^[5]^ Therefore, using the SEER database to construct a corresponding nomogram model to clarify the long-term complications and survival prognosis at the population level of lung cancer patients is the focus of this study, in order to provide some reference for future clinical issues regarding tertiary prevention and survival prediction of multiple primary cancers.

## 2. Data and Methods

### 2.1. Case collections

In this study, data on patients diagnosed with lung cancer from 2010 to 2015 were retrieved from the SEER-18 database (covering approximately 28% of the US population) using SEER* Stat software (version 8.4.0.1). Extracted information mainly included demographic characteristics, histology type, treatment modality, and other relevant information. Specific indicators included age at diagnosis, race, gender, marital status, and histological type, site of lesion, degree of differentiation, TNM stage, treatment (surgery, lymph node dissection, radiotherapy, chemotherapy), and survival time for first primary cancer (FPC) and second primary cancer (SPC).

Inclusion criteria: the FPC was lung cancer, double primary cancer, meeting the primary tumor criteria, and age at diagnosis of first primary lung cancer (FPLC) older than or equal to 18 years.

Exclusion criteria: patients with missing or incomplete clinical data, such as race, marital status, survival time of 0 or failure to follow-up; and 3 or more primary cancers.

The final study cohort was randomized 7:3 into the modeling and validation groups.

### 2.2. Statistical analysis

This study was statistically analyzed using SPSS software (version 26. 0, IBM, USA) and R language (version 4.1.0). The hazard ratio and 95% confidence interval were derived from univariate and multivariate COX risk model analysis to identify independent prognostic factors. The accuracy of the nomogram was evaluated using the area under the curve (AUC) of the receiver operating characteristic. The value of clinical application was evaluated by the calibration curve. Survival curves were plotted by the Kaplan–Meier (K-M) method for survival analysis. It was considered statistically significant at a *P* value of < .05.

## 3. Results

### 3.1. Patient clinical characteristics

We included a total of 9306 patients: 6516 patients in the modeling group and 2790 patients in the validation group. We mapped the demographic characteristics and basic clinical features of the patients, as shown in Table 1. When we retrieved that FPC was lung cancer, we found that the most common site of SPC was located in the respiratory system, followed by the genitourinary and digestive systems, with partial involvement of various organs in the head and neck. Among them, the respiratory system was as high as 54.0%. In addition, we summarized the sites of the first primary lung cancer in patients with double primary cancer accordingly and found that the most common site was the right upper lobe (33.3%), followed by the left upper lobe, right lower lobe, left lower lobe, and right middle lobe.

**Table 1:** Demographic and clinical characteristics of the patients.

Clinical features	The modeling group		The validation group		Total		*P*
	No. (n)	Percentage	No. (n)	Percentage	No. (n)	Percentage	
Age* (yr)							**0.332**
<60	1130	17.3	449	16.1	1579	17.0	
60–69	2327	35.7	1037	37.2	3364	36.1	
70–79	2261	34.7	951	34.1	3212	34.5	
≥80	798	12.2	353	12.7	1151	12.4	
Gender							**0.897**
Male	3496	53.7	1501	53.8	4997	53.7	
Female	3020	46.3	1289	46.2	4309	46.3	
Race							**0.410**
White	5399	82.9	2305	82.6	7704	82.8	
Black	706	10.8	323	11.6	1029	11.1	
Other	411	6.3	162	5.8	573	6.2	
Marital status							**0.712**
Married	3665	56.2	1535	55.0	5200	55.9	
Divorced	849	13.0	378	13.5	1227	13.2	
Single	859	13.2	370	13.3	1229	13.2	
Other	1143	17.5	507	18.2	1650	17.7	
Histology*							**0.590**
SCLC	343	5.3	154	5.5	497	5.3	
LUSC	1806	27.7	804	28.8	2610	28.0	
LUAD	3345	51.3	1415	50.7	4760	51.1	
Other	1022	15.7	417	14.9	1439	15.5	
Site*							**0.055**
Upper left lobe	1700	26.1	780	28.0	2480	26.6	
Lower left lobe	932	14.3	353	12.7	1285	13.8	
Upper right lobe	2171	33.3	927	33.2	3098	33.3	
Middle right lobe	326	5.0	116	4.2	442	4.7	
Lower right lobe	1003	15.4	457	16.4	1460	15.7	
Other	384	5.9	157	5.6	541	5.8	
Grade*							**0.713**
High differentiation	694	10.7	291	10.4	985	10.6	
Middle differentiation	1866	28.6	794	28.5	2660	28.6	
Low differentiation	1756	26.9	767	27.5	2523	27.1	
Undifferentiated	145	2.2	50	1.8	195	2.1	
Unknown	2055	31.5	888	31.8	2943	31.6	
Stage*							**0.920**
I	2955	45.3	1258	45.1	4213	45.3	
II	960	14.7	422	15.1	1382	14.9	
III	1413	21.7	593	21.3	2006	21.6	
IV	1188	18.2	517	18.5	1705	18.3	
Surgery*							**0.843**
No	3164	48.6	1361	48.8	4525	48.6	
Yes	3352	51.4	1429	51.2	4781	51.4	
LN*							**0.507**
No	2999	46.0	1305	46.8	4304	46.2	
Yes	3517	54.0	1485	53.2	5002	53.8	
Radiotherapy*							**0.791**
No	4043	62.0	1723	51.8	5766	62.0	
Yes	2473	38.0	1067	38.2	3540	38.0	
Chemotherapy*							**0.956**
No	3943	60.5	1690	60.6	5633	60.5	
Yes	2573	39.5	1100	39.4	3673	39.5	
Site†							**0.481**
R	3518	54.0	1508	54.1	5026	54.0	
HN	455	7.0	197	7.1	652	7.0	
D	1010	15.5	427	15.3	1437	15.4	
G	1158	17.8	521	18.7	1679	18.0	
Other	375	5.8	137	4.9	512	5.5	
Grade†							**0.613**
High differentiation	576	8.8	250	9.0	826	8.9	
Middle differentiation	1248	19.2	550	19.7	1798	19.3	
Low differentiation	1027	15.8	415	14.9	1442	15.5	
Undifferentiated	247	3.8	99	3.5	346	3.7	
Other (B cell, T cell)	43	0.7	12	0.4	55	0.6	
Unknown	3375	51.8	1464	52.5	4839	52.0	
Stage†							**0.725**
0	250	3.8	126	4.5	376	4.0	
I	1899	29.1	818	29.3	2717	29.2	
II	563	8.6	245	8.8	808	8.7	
III	576	8.8	235	8.4	811	8.7	
IV	731	11.2	307	11.0	1038	11.2	
Unknown	2497	38.3	1059	38.0	3556	38.2	
Surgery†							**0.619**
No	4006	61.5	1700	60.9	5706	61.3	
Yes	2510	38.5	1090	39.1	3600	38.7	
LN†							**0.790**
No	4954	76.0	2114	75.8	7068	76.0	
Yes	1562	24.0	676	24.2	2238	24.0	
Radiotherapy†							**0.343**
No	4386	67.3	1906	68.3	6292	67.6	
Yes	2130	32.7	884	31.7	3014	32.4	
Chemotherapy†							**0.673**
No	4779	73.3	2058	73.8	6837	73.5	
Yes	1737	26.7	732	26.2	2469	26.5	
Surgery*,†							**0.818**
No/no	2409	37.0	1040	37.3	3449	37.1	
No/yes	755	11.6%	321	11.5	1076	11.6	
Yes/no	1597	24.5	660	23.7	2257	24.3	
Yes/yes	1755	26.9	769	27.6	2524	27.1	
LN*,†							**0.720**
No/no	2635	40.4	1153	41.3	3788	40.7	
No/yes	364	5.6	152	5.4	516	5.5	
Yes/no	2319	35.6	961	34.4	3280	35.2	
Yes/yes	1198	18.4	524	18.8	1722	18.5	
Radiotherapy*,†							**0.594**
No/no	3020	46.3	1316	47.2	4336	46.6	
No/yes	1023	15.7	407	14.6	1430	15.4	
Yes/no	1366	21.0	590	21.1	1956	21.0	
Yes/yes	1107	17.0	477	17.1	1584	17.0	
Chemotherapy*,†							**0.953**
No/no	3224	49.5	1382	49.5	4606	49.5	
No/yes	719	11.0	308	11.0	1027	11.0	
Yes/no	1555	23.9	676	24.2	2231	24.0	
Yes/yes	1018	15.6	424	15.2	1442	15.5	

D = digestive system, G = genitourinary system, HN = head and neck, LN = lymph node dissection, LUAD = lung adenocarcinoma, LUSC = lung squamous carcinoma, R = respiratory system, SCLC = small cell lung cancer.

* First primary cancer.

† Second primary cancer.

### 3.2. Univariate and multivariate prognostic analysis

In this study, we identified the following clinical characteristics as prognostic factors for SPC: age, sex, race, and marital status, as well as histological type, lesion site, degree of differentiation, TNM stage, and treatment modality (surgery, lymph node dissection, radiotherapy, and chemotherapy) for both FPC and SPC, as shown in Table 2. In addition, multivariate COX analysis identified 14 independent prognostic factors associated with secondary SPC in patients with lung cancer.

**Table 2:** Univariate and multivariate Cox analysis of prognosis in patients with double primary carcinoma in which lung cancer was the first primary cancer (the modeling group).

Clinical features	Univariate analysis			Multivariate analysis		
	HR	95% CI	*P*	HR	95% CI	*P*
Age* (yr)						**<0.001**
<60	Reference			Reference		
60–69	0.988	0.884–1.104	0.833	1.076	0.961–1.205	0.205
70–79	1.205	1.079–1.345	**0.001**	1.332	1.187–1.495	**<0.001**
≥80	1.505	1.314–1.724	**<0.001**	1.492	1.288–1.729	**<0.001**
Gender						**<0.001**
Male	Reference			Reference		
Female	0.747	0.693–0.805	**<0.001**	0.742	0.684–0.804	**<0.001**
Race						**0.002**
White	Reference			Reference		
Black	1.137	1.013–1.276	**0.029**	0.961	0.852–1.084	0.520
Other	0.920	0.786–1.076	0.296	0.745	0.634–0.875	**<0.001**
Marital status						**0.007**
Married	Reference			Reference		
Divorced	1.041	0.927–1.169	0.494	1.114	0.989–1.255	0.075
Single	1.167	1.045–1.304	**0.006**	1.124	1.003–1.261	**0.045**
Other	1.213	1.098–1.340	**<0.001**	1.184	1.063–1.318	**0.002**
Histology*						**<0.001**
SCLC	Reference			Reference		
LUSC	0.593	0.512–0.688	**<0.001**	0.974	0.826–1.147	0.749
LUAD	0.408	0.354–0.471	**<0.001**	0.820	0.699–0.963	**0.015**
Other	0.522	0.443–0.614	**<0.001**	0.750	0.631–0.893	**0.001**
Site*						0.089
Upper left lobe	Reference			Reference		
Lower left lobe	0.962	0.850–1.088	0.534	0.971	0.857–1.100	0.642
Upper right lobe	0.964	0.874–1.062	0.455	0.944	0.856–1.042	0.256
Middle right lobe	0.746	0.613–0.908	**0.003**	0.809	0.663–0.986	0.036
Lower right lobe	1.006	0.893–1.132	0.926	1.077	0.955–1.214	0.229
Other	1.639	1.405–1.912	**<0.001**	0.992	0.846–1.164	0.922
Grade*						**<0.001**
High differentiation	Reference			Reference		
Middle differentiation	1.660	1.408–1.957	**<0.001**	1.435	1.212–1.699	**<0.001**
Low differentiation	2.151	1.827–2.533	**<0.001**	1.503	1.267–1.783	**<0.001**
Undifferentiated	2.868	2.192–3.753	**<0.001**	1.660	1.245–2.213	**0.001**
Unknown	3.240	2.762–3.801	**<0.001**	1.375	1.157–1.635	**<0.001**
Stage*						**<0.001**
I	Reference			Reference		
II	1.470	1.303–1.657	**<0.001**	1.563	1.376–1.775	**<0.001**
III	2.348	2.127–2.591	**<0.001**	2.001	1.770–2.262	**<0.001**
IV	4.754	4.314–5.239	**<0.001**	3.123	2.749–3.547	**<0.001**
Site†						**<0.001**
R	Reference			Reference		
HN	1.184	1.025–1.369	**0.022**	1.125	0.967–1.309	0.127
D	0.975	0.869–1.093	0.664	0.906	0.802–1.023	0.113
G	0.846	0.761–0.940	**0.002**	0.717	0.633–0.812	**<0.001**
Other	0.591	0.486–0.719	**<0.001**	0.623	0.501–0.775	**<0.001**
Grade†						**0.007**
High differentiation	Reference			Reference		
Middle differentiation	1.420	1.198–1.682	**<0.001**	1.245	1.048–1.480	**0.013**
Low differentiation	1.765	1.488–2.094	**<0.001**	1.354	1.135–1.615	**0.001**
Undifferentiated	1.687	1.325–2.147	**<0.001**	1.496	1.164–1.922	**0.002**
Other (T cell, B cell)	0.735	0.376–1.435	0.366	0.957	0.472–1.938	0.902
Unknown	1.631	1.398–1.903	**<0.001**	1.313	1.117–1.544	**0.001**
Stage†						**<0.001**
0	Reference			Reference		
I	1.091	0.895–1.331	0.388	1.022	0.829–1.261	0.838
II	1.131	0.903–1.415	0.284	1.022	0.807–1.295	0.854
III	1.674	1.349–2.077	**<0.001**	1.285	1.020–1.619	**0.034**
IV	2.692	2.187–3.313	**<0.001**	1.435	1.144–1.799	**0.002**
Unknown	0.591	0.484–0.722	**<0.001**	0.492	0.398–0.609	**<0.001**
Surgery*,†						**<0.001**
No/no	Reference			Reference		
No/yes	0.600	0.532–0.676	**<0.001**	0.615	0.531–0.712	**<0.001**
Yes/no	0.331	0.300–0.365	**<0.001**	0.586	0.498–0.691	**<0.001**
Yes/yes	0.235	0.212–0.262	**<0.001**	0.348	0.290–0.417	**<0.001**
LN*,†						**<0.001**
No/no	Reference			Reference		
No/yes	0.644	0.548–0.756	**<0.001**	0.796	0.666–0.952	**0.012**
Yes/no	0.373	0.342–0.406	**<0.001**	0.792	0.697–0.900	**<0.001**
Yes/yes	0.329	0.293–0.370	**<0.001**	0.774	0.657–0.911	**0.002**
Radiotherapy*,†						**<0.001**
No/no	Reference			Reference		
No/yes	0.661	0.585–0.747	**<0.001**	0.593	0.519–0.678	**<0.001**
Yes/no	1.571	1.433–1.724	**<0.001**	0.814	0.730–0.909	**<0.001**
Yes/yes	1.336	1.206–1.480	**<0.001**	0.549	0.487–0.619	**<0.001**
Chemotherapy*,†						**<0.001**
No/no	Reference			Reference		
No/yes	0.941	0.823–1.077	0.379	0.888	0.772–1.023	0.100
Yes/no	1.435	1.310–1.572	**<0.001**	0.764	0.681–0.856	**<0.001**
Yes/yes	2.019	1.829––2.228	**<0.001**	0.830	0.736––0.935	**0.002**

95% CI = 95% confidence interval, D = digestive system, G = genitourinary system, HN = head and neck, HR = hazard ratio; LN = lymph node dissection, LUAD = lung adenocarcinoma, LUSC = lung squamous carcinoma, R = respiratory system, SCLC = small cell lung cancer.

* First primary cancer.

† Second primary cancer.

### 3.3. Construction and validation of prediction models

**Figure 1.**
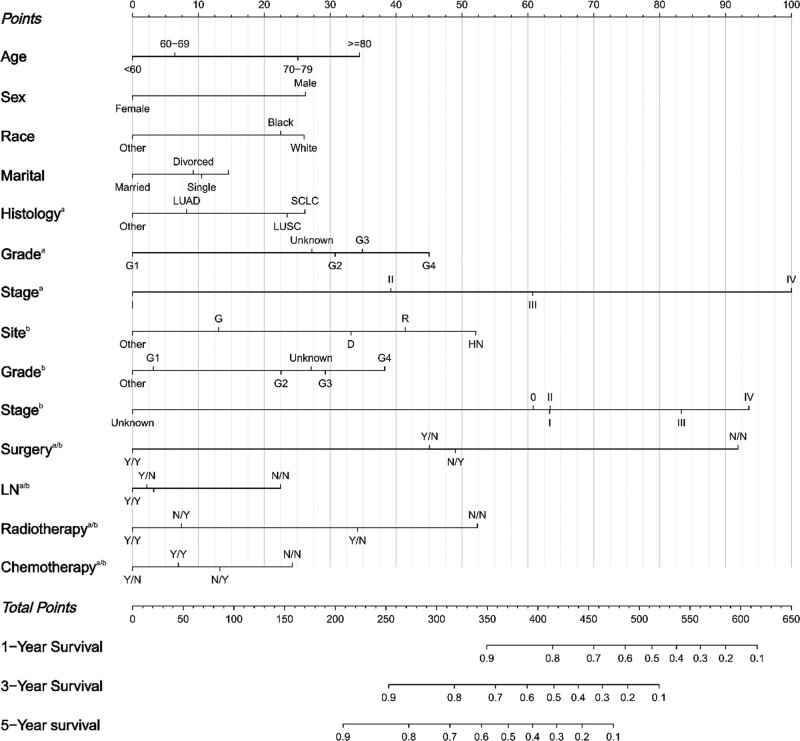
Survival nomogram for prognosis of double primary carcinoma with lung cancer as the first primary cancer. According to a multivariate Cox regression analysis, we ascertained the independent prognostic factors (age, sex, race, marital, histology^a^, grade^a^, stage^a^, site^b^, grade^b^, stage^b^, surgery^a/b^, LN^a/b^, radiotherapy^a/b^, and chemotherapy^a/b^) predicting survival outcomes in patients with first diagnosed lung cancer recurrent second primary cancer. The corresponding 1-, 3-, and 5-yr survival rates for a particular patient with this disease are obtained by summing the scores of the corresponding variables for the individual patient, finding the corresponding total score on the total point axis, and drawing a line downward. a = first primary cancer, b = second primary cancer. D = digestive system, G = genitourinary system, HN = head and neck, LN = Lymph node dissection, LUAD = lung adenocarcinoma, LUSC = lung squamous carcinoma, N = no, R = respiratory system, SCLC = small cell lung cancer, Y = yes.

**Figure 2.**
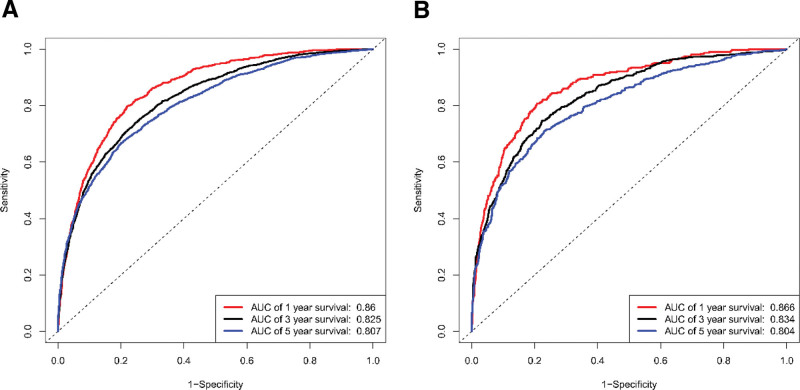
Receiver operating characteristic curves for modeling group (A) and validation group (B).

**Figure 3.**
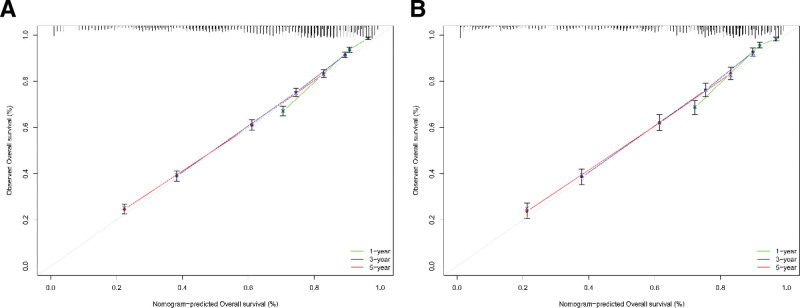
Calibration diagrams for modeling group (A) and verification group (B).

Based on 14 independent risk factors revealed by multivariate COX analysis, we developed a nomogram model for predicting survival outcomes in patients with first diagnosed lung cancer recurrent SPC (Fig. 1). The results showed that the tumor stage of first diagnosed lung cancer contributed the most to the prognosis. The area under the receiver operating characteristic curve for the modeling group AUC = 0.86, 0.825, and 0.807 (1-, 3-, and 5-year survival, respectively), indicated that the model had high predictive accuracy (Fig. 2A). Meanwhile, the corresponding calibration curve was plotted according to the predicted and actual patient survival. Where, the vertical and horizontal coordinates represent the actual and predicted survival probabilities of the model, respectively. The results showed that the calibration curves of the modeling group had good clinical applicability (Fig. 3A). This research team utilized the validation group for the validation of the model. The results showed that, the AUC of the validation cohort model also exceeded 0.75, and the calibration curve also showed a good linear relationship (Figs. 2B and 3B).

### 3.4. Survival analysis

**Figure 4.**
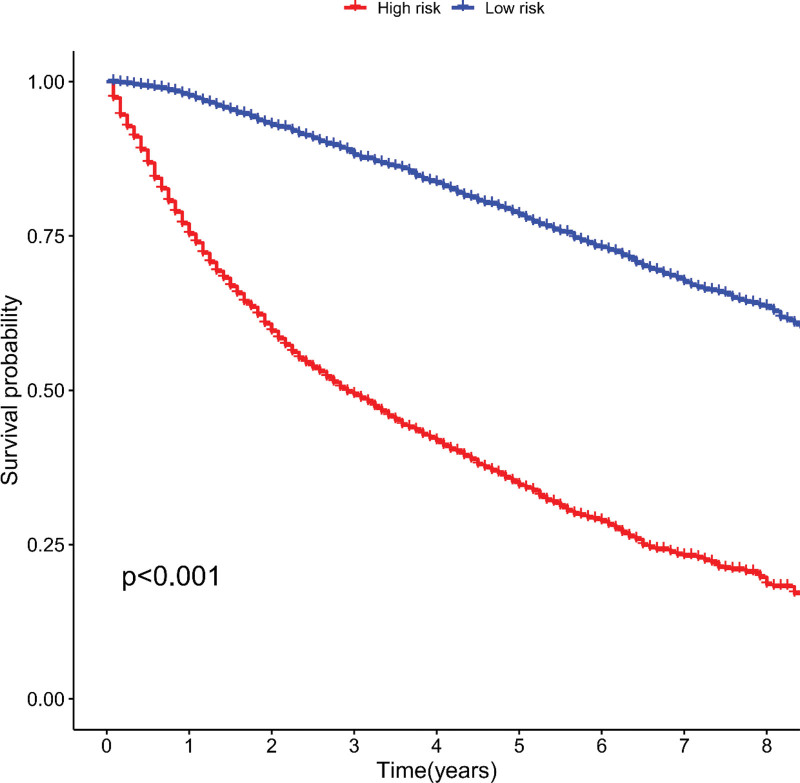
Effect of different risk levels on cancer-specific survival.

We scored the independent prognostic factors included in the multifactorial Cox regression analysis accordingly, with a final score above the median being judged as high risk and vice versa. The final risk score results were derived and survival curves were plotted using the K-M method to compare cancer-specific survival between different risk subgroups (Fig. 4).

## 4. Discussion

As a major lung cancer country, China had about 820,000 new lung cancer cases and 710,000 lung cancer deaths in 2020.^[1]^ In recent years, with the change in the patients’ lifestyle, improvement of compliance and development of imaging technology, the detection rate of patients with double primary cancer has gradually increased, which has attracted the attention of clinicians. However, a large number of previous studies have mostly focused on single primary lung cancer and multiple primary lung cancer.^[6–9]^ There are fewer studies on SPC about lung cancer combined with SPC, and it is easily confused with metastatic lung cancer, leading to loss of patient confidence in the treatment and poor prognosis. Therefore, we retrospectively collected a total of 9306 SPC patients whose first cancer was lung cancer from 2010 to 2015 in the SEER database for clinical analysis, and aimed to raise clinicians’ awareness by developing predictive models related to the prognosis of such diseases. We hope that the results of our study will help clinical practitioners to focus on the identification of multiple primary cancers and prolong the survival time of patients.

In this study, a survival nomogram model for predicting prognosis associated with this type of disease was developed by assessing prognostic factors associated with patients with double primary carcinoma of the first diagnosis of lung cancer. Identifying the common sites of secondary SPC after the occurrence of lung cancer is clinically important to improve the effectiveness and aggressiveness of follow-up of oncology patients. We ultimately included 9306 patients and identified corresponding independent prognostic factors including age, sex, race, and marital status, as well as histological type, lesion site, degree of differentiation, TNM stage, and treatment modality (surgery, lymph node dissection, radiotherapy, and chemotherapy). When we retrieved that the FPC was lung cancer, we found that the site of occurrence was mostly in the right upper lobe (33.3%) and the most common site of SPC was in the respiratory system (54.0%).

In addition, understanding the treatment options for patients with dual primary cancers helps clinicians to individualize their treatment and follow-up plans. In the study of risk factors for multiple primary cancers, it was found that radiotherapy and chemotherapy for the first cancer may contribute to the development of SPC.^[3]^ This is consistent with our study, which showed that surgery and lymph node dissection of the primary site was a relatively protective factor for patients, while radiotherapy and chemotherapy to the primary site was a relative risk factor.

Of course, there are still some limitations to this study. First, this study is based on the SEER database, which cannot analyze certain common risk factors, such as smoking, alcohol consumption status, genetic information.^[10]^ Second, this study did not provide in-depth analysis of the patients’ treatment modalities, such as surgical modality, radiotherapy modality (e.g., stereotactic radiation therapy, tumor ablation, etc), chemotherapy modality, drug selection, etc, which still need further study. In addition, the SEER database is an open database established by the National Institutes of Research, and the group studied is predominantly White, lacking our domestic data. Regarding our research in this area, further multicenter and prospective studies by researchers and clinical workers in various fields may be needed to improve our prognostic risk prediction model. Notably, this study also lacks the analysis of factors related to the development of such disease and a novel nomogram model for predicting SPC, which needs to be gradually improved in our next work and provides some reference for the development of a better diagnosis and follow-up strategy.

In conclusion, this study constructs a prognostic model for patients with second primary carcinoma secondary to first diagnosed lung cancer with high accuracy and clinical applicability. With the number of patients with multiple primary cancers detected increasing year-by-year, clinical workers should pay more attention to such diseases. Early detection and diagnosis as well as early treatment will greatly improve the survival time and survival quality of such patients.

## Author contributions

**Conceptualization:** Bin Hu.

**Data curation:** Bin Hu, Wanjiao Chen, Jiarong Lv.

**Formal analysis:** Bin Hu, Ningjie Xu.

**Funds:** Shifang Sun.

**Methodology:** Wanjiao Chen, Jiarong Lv.

**Supervision:** Shifang Sun.

**Writing—original draft:** Bin Hu.

**Writing—review and editing:** Shifang Sun, Yifeng Mai.

**Writing—revision:** Bin Hu, Wanjiao Chen, Ningjie Xu.
